# Towards efficient and accurate prediction of freeway accident severity using two-level fuzzy comprehensive evaluation

**DOI:** 10.1016/j.heliyon.2024.e36396

**Published:** 2024-08-22

**Authors:** Guanghui Wang, Jinbo Li, Lingfeng Shen, Shuang Ding, Zongqi Shi, Fang Zuo

**Affiliations:** a School of Software, Henan University, Kaifeng, 475004, China; b Henan International Joint Laboratory of Intelligent Network Theory and Key Technology, Henan University, Kaifeng, 475004, China; c Henan Communications Planning & Design Institute Co. Ltd, Zhengzhou, 450008, China

**Keywords:** Freeway accidents, Severity prediction, Two-level fuzzy comprehensive evaluation

## Abstract

Accurately predicting freeway accident severity is crucial for accident prevention, road safety, and emergency rescue services in intelligent freeway systems. However, current research lacks the required precision, hindering the effective implementation of freeway rescue. In this paper, we efficiently address this challenge by categorizing influencing factors into two levels: human and non-human, further subdivided into 6 and 36 categories, respectively. Furthermore, based on the above factors, an efficient and accurate Freeway Accident Severity Prediction (FASP) method is developed by using the two-level fuzzy comprehensive evaluation. The factor and evaluation sets are determined by calculating the fuzzy evaluation matrix of a single factor. The weight matrix is calculated through the entropy method to compute the final evaluation matrix. Based on the maximum membership principle, the severity of the freeway accident is predicted. Finally, based on the experiments conducted with the traffic accident datasets in China and the US, it is shown that FASP is able to accurately predict the severity of freeway traffic accidents with thorough considerations and low computational cost. It is noted that FASP is the first attempt to achieve freeway accident severity prediction using the two-level fuzzy comprehensive evaluation method to the best of our knowledge.

## 1 Introduction

Intelligent freeway systems are a collection of advanced technologies and communication networks used in freeway infrastructure to improve traffic flow, enhance safety, reduce congestion, and increase efficiency and sustainability. Rapid economic growth has determined significant changes in its transportation and infrastructure. From a transportation perspective, the number of vehicles has grown dramatically across each country [1], [2]. Road safety has become a worldwide problem due to the serious consequences caused by road traffic accidents [3], [4], [5]. Approximately 1.30 million people die each year in road traffic accidents, the leading cause of death among children and young people today [6]. The vast majority of these accidents are caused by human error [7], [8]. There were 247,646 traffic accidents with 62,763 fatalities and direct property damage of 1.46 billion Yuan nationwide [9], and the percentage increase in direct property damage due to traffic accidents was 24.8% in 2019 compared to 2011 [10], [11].

Freeway traffic accidents become severe and have a considerable negative impact on economic growth, public health, and social welfare. A study from Tsinghua University showed that while the total number of road traffic accidents in China decreased, accident severity increased [12]. Another study noted that motor vehicle fatalities decreased, while non-motor vehicle fatalities increased [13]. There are several factors that can contribute to the severity of freeway traffic accidents, including higher speeds, distraction, impaired driving, aggressive driving, lack of seat belt usage, and road conditions. Therefore, it is important to study the severity of freeway traffic accidents to minimize the risk of accidents and mitigate their severity [14], [15].

Predicting the severity of freeway traffic accidents is an important way to prevent freeway traffic accidents and ensure the safety of road users [16]. The prediction of freeway accident severity usually involves using statistical models and machine learning techniques to estimate the severity of accidents on freeways. By analyzing and evaluating various factors that occur during accidents, accident severity prediction is beneficial for taking corresponding measures to reduce casualties and losses. Firstly, accident severity prediction can help emergency responders make more accurate decisions. After an accident, emergency personnel need to quickly understand the severity of the accident in order to determine whether additional rescue resources and personnel need to be dispatched. By accurately predicting the severity of accidents, emergency responders can better allocate resources, provide timely and appropriate medical treatment, and save lives to the greatest extent possible [17]. Secondly, accident severity prediction is crucial for traffic management authorities. By analyzing accident data and related factors, we can identify high-risk areas and accident-prone locations. Traffic management authorities can use this information to develop targeted traffic planning and safety measures, such as increasing traffic signals, installing speed bumps, improving road conditions, and thereby reducing the severity of accidents [18]. Consequently, it is significant to predict the severity of freeway accidents for emergency response and traffic management, and it helps take appropriate actions to maximize the protection of people's lives and property.

Existing methods usually use machine learning to predict the severity of freeway accidents [14], [19]. The reference [20] utilizes thirteen common machine learning algorithms to predict the severity of accidents. Random forest and Bayesian optimization techniques were applied for freeway accident prediction [21], [22], [23]. Accident severity was predicted by multiple logistic regression models, considering various individual influencing variables [24], [23]. Although there are several methods and tools available for predicting freeway accident severity, accurately predicting the severity of accidents can still be challenging [25], [26]. It involves complex variables and factors, such as weather conditions, road infrastructure, traffic volume, driver behavior, and vehicle characteristics. Obtaining comprehensive and reliable data on accidents, traffic volume, and other relevant factors can be difficult. Freeway traffic conditions can change rapidly and predictive models need to be updated frequently to reflect changing conditions. Additionally, driver behavior is unpredictable and difficult to model, making it hard to predict how drivers will react in different situations. Severe accidents are relatively rare events, and it is challenging to capture enough data on them to build reliable predictive models. Existing machine learning methods, such as DNN and random forest, often reduce the dimensionality of features to simplify the model and speed up calculations. However, the use of dimensionality reduction technology ignores the impact of some accident severity, leading to a lack of accuracy in predicting the severity of the accident. Existing non-machine learning methods do not have the problem of reducing the dimensionality of some influencing factors when predicting the severity of the accident. However, these methods consider fuzzy relationships among factors that are not comprehensive enough, resulting in a need to improve the accuracy of predicting accident severity. Therefore, while technology has advanced significantly in this area, there is still room for improvement in order to provide more accurate and reliable predictions on the severity of freeway accidents [27].

In this paper, we propose an efficient and accurate Freeway Accident Severity Prediction (FASP) method by using the two-level fuzzy comprehensive evaluation. The severity of three types of freeway accidents (i.e., death, injuries, and property damage) is efficiently and accurately predicted. The factors affecting freeway accidents are collected and divided into two levels. According to whether people are involved, the first level factors are divided into human factors and non-human factors. In the second level factors, the human factors and non-human factors are further divided into 6 and 36 factors, respectively. Besides, the factor and evaluation sets are determined to calculate the fuzzy evaluation matrix of a single factor. The weight matrix is calculated through the entropy method to compute the final evaluation matrix for the factors. Based on the maximum membership principle, the severity of the freeway accident is predicted. Experiments on Chinese traffic accident datasets show that the developed FASP method accurately and efficiently predicts the severity of freeway traffic accidents. More specifically, our contributions are summarized as follows:

• We investigate a novel problem of efficiently and accurately predicting the severity of freeway accidents. It is the first attempt to achieve freeway accident severity prediction using the two-level fuzzy comprehensive evaluation method to the best of our knowledge.
• We propose an efficient and accurate Freeway Accident Severity Prediction (FASP) method. The FASP method fully considers the factors that affect accident severity and the fuzzy relationship between factors, which makes the method more accurate. The fuzzy operations ensure the efficiency of the FASP method.
• We conduct experiments with Chinese traffic accident datasets and US traffic accident datasets. The results show that the developed FASP method is able to accurately predict the severity of freeway traffic accidents with comprehensive considerations and low computational cost.

The rest of the paper is organized as follows. In Section 2, the research progress of freeway accident severity prediction and fuzzy mathematics in recent years is summarized. Section 3 is the system method and problem description. Section 4 describes the system architecture and the concrete steps of the proposed method. Section 5 is the experimental setup and results. Section 6 concludes the paper.

## 2 Related work

In this section, we first summarize the literature on freeway accident severity prediction. Next, we review the two-level fuzzy comprehensive evaluation method.

### 2.1 Freeway accident severity prediction

There are many studies on freeway accident severity prediction. The first DNN-based multi-task model for predicting freeway accident severity was proposed in Reference [14]. The reference [28] for the first time utilized RF, GB, and SVM to analyze the severity of vehicle-to-vehicle collisions among drivers in the United Arab Emirates. A method for predicting the severity of traffic accidents was proposed based on decision-level fusion of machine and deep learning models [19]. In a hybrid model that combines random forest and Bayesian optimization, promising outcomes were observed in the prediction of freeway accidents [21]. Previous studies introduced a novel framework for predicting traffic accidents [29]. This framework incorporates a two-stream network that utilizes a two-layer hidden state aggregation technique. Besides, a state-of-the-art technique was proposed for the detection of freeway accident severity, utilizing the power of convolutional neural networks. This pioneering method demonstrated significant potential in advancing the accuracy and efficiency of accident severity detection [30]. A predictability model was employed to depict the relationship between road hazards and relevant constraints. Multiple linear regression and artificial neural network prediction models were compared in their study [16]. The reference [31] proposed a two-layer stacking model, EnLKtreeGBDT, based on semantic understanding for predicting the severity of traffic accidents. This model used semantic enhancement and data augmentation modules to improve prediction accuracy. Machine learning algorithms, econometric techniques, and traditional statistical methods were combined to analyze and predict the severity of road traffic accidents in the UK [32]. Machine learning and deep learning algorithms were subjected to cognitive analysis to identify the main factors affecting the severity of traffic accidents in India [33]. The reference [34] compared the performance of statistical models and machine learning models in classifying accident severity, demonstrating that for extremely imbalanced small sample data, the performance of machine learning models was relatively superior. A deep forest algorithm was proposed for predicting the severity of traffic accidents. Experiments showed that this method has good stability, fewer hyperparameters, and achieved the highest accuracy across different training data sizes [35]. Yang et al. innovatively modified commonly used accident features in previous studies, utilizing a random forest model to predict the severity of car accidents in China from 2018 to 2020, resulting in a highly accurate prediction model [36]. Wang et al. used traffic accident data from Shenyang, Liaoning Province, China, as the research object, combining random forest and association rule algorithms to explore the risk factors affecting the severity of traffic accidents [37]. Ceven et al. studied urban traffic accident report data from Turkey, using ensemble learning methods such as random forest, AdaBoost, and multilayer perceptron to conduct a three-level classification prediction of traffic accident severity, identifying the main factors influencing accident severity [38]. The aforementioned machine learning algorithms have shown good accuracy in accident prediction. However, when using machine learning methods for prediction, dimensionality reduction is often applied to reduce the dimensionality of the feature space, simplifying the model and improving computational speed. The process of dimensionality reduction may overlook important feature information, leading to a decrease in model accuracy. Additionally, there can be uneven data distribution, which further reduces accuracy when predicting the severity of data with less occurrence.

The proportion of accidental property damage attributed to road infrastructure damage is investigated for freeway accident severity prediction. A study was conducted employing a Bayesian stochastic parametric Tobit model [22]. The contribution of [39] is the incorporation of crash severity into hot spot analysis using GIS, which could lead to better-informed decision-making in the realm of highway safety. Different individual influencing variables were analyzed by multivariate logistic regression models to predict the severity of accidents [24]. The frequency and severity of the accident model are estimated by analyzing and classifying homogeneous segments using a spatial approach and the generalized estimating equation model [40]. A framework to discover interpretable regression models was introduced by clustering the importance of features from a post hoc interpretable framework into a highly flexible predictive model [41]. To predict traffic accidents, a novel data-driven model has been proposed. This model is built upon an extended belief rule-based system and takes into account the enhancement of traffic safety efficiency [11]. [42] uses a hybrid analytic hierarchy process (AHP) and the preference ranking organization method for enrichment evaluation (PROMETHEE) approach to analyze the severity of factors and characteristics that influence road accidents. However, that method did not compare the results with traditional machine learning methods, and part of the process requires expert evaluation, which introduces a certain level of subjectivity. Bermudez et al. aimed to determine to what extent these measures have achieved their intended goals by analyzing traffic accident data, thereby providing a basis and recommendations for formulating similar policies in future [43]. The above-mentioned work included non-machine learning methods to predict the severity of accidents not involving dimensionality reduction. However, these methods do not consider the fuzzy relationships between factors. They may still have lower prediction accuracy for accidents with lower data distribution.

### 2.2 Two-level fuzzy comprehensive evaluation

The two-level fuzzy comprehensive evaluation is based on fuzzy mathematics and derived from fuzzy comprehensive evaluation [44], [45]. Many important factors impact the priorities of alternatives, for instance, the weights of attributes or criteria, attitudinal character [46]. On the other hand, for groundwater health risk assessment, a fuzzy comprehensive evaluation was performed using the analytic hierarchy process and entropy method [47]. A vulnerability evaluation of the marine economic system based on a fuzzy comprehensive evaluation model was conducted in Reference [48]. The ecosystem health evaluation of a desert nature reserve was conducted, using entropy power and fuzzy mathematics as the assessment methods [49]. Fuzzy mathematics was employed as a comprehensive approach to decipher the forest community environment of a mountain [50]. Furthermore, comprehensive fuzzy evaluation is widely used in the fields of medicine and habitat suitability analysis [51], [52], [53]. The comprehensive benefits of land use were evaluated using fuzzy mathematics and biological heuristics, considering the inputs and outputs of land [54]. The aforementioned paper has extensively applied fuzzy comprehensive evaluation in various fields. However, its application is relatively limited for the freeway accident severity prediction in the transportation domain.

There are also some related works that, although not directly using fuzzy comprehensive evaluation, have extensively applied fuzzy theory and membership functions in various fields such as sensory evaluation of liquor, spatial distribution patterns of water pollutants, and safety of gas tunnel concrete structures. Fuzzy mathematics methods were applied to identify the optimal process parameters for peanut sprout yogurt, considering its fundamental sensory indicators [55]. Fuzzy mathematics methods and principal component analysis were applied to comprehensively analyze the sensory evaluation and physicochemical indicators of diverse strong-aroma Baijiu liquors. This analysis enabled a quantitative assessment of their sensory quality [56]. The spatial distribution patterns of water pollutants were summarized using the comprehensive evaluation method of fuzzy mathematics [57]. In addition, an assessment of the safety of gas tunnel concrete structures was conducted based on the theory of fuzzy mathematics [58]. Fuzzy membership functions, the maximum entropy principle, fuzzy mathematics for comprehensive evaluation, and the Bayesian network framework were implemented to simulate the correlation between species habitat and environmental variables [53]. For exploring the essence of industrial design in relation to the adaptability of sports equipment to the human body, fuzzy mathematics theory was employed as an analytical tool [59]. An investigation was undertaken to quantify the influencing factors of key monitoring indicators in the finished oil market and visualize them using fuzzy mathematics methods and big data analysis techniques [60]. Fault Tree Analysis combined with fuzzy mathematics was used to analyze accidents in the railway hazardous goods transportation system [61]. Therefore, the fuzzy comprehensive evaluation provides a promising way to predict the freeway accident severity.

## 3 System model and problem description

### 3.1 System model

**Figure 1:**
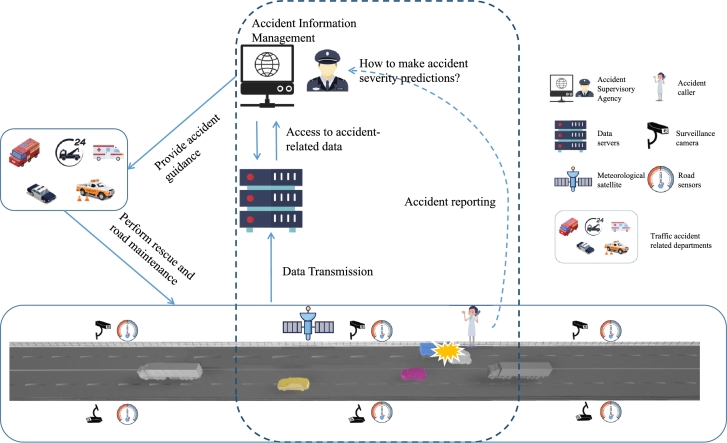
System model for the freeway accident severity prediction.

The system model is described for the freeway accident severity prediction. Freeway traffic accidents occur frequently, resulting in injuries or even fatalities for drivers and passengers, while also causing significant property and economic losses. As shown in Fig. 1, the dashed box represents the focal point of the research. Cameras and road condition sensors are installed near the freeway. The aforementioned edge devices continuously update weather and road data to a data server in real time. When a traffic accident occurs on the freeway, the accident is reported through mobile phones, including information such as the location and extent of injuries, to the data server. The freeway accident regulatory department retrieves accident-related data from the data server through the accident regulatory host. The key issue lies in how to use the data to make predictions about the severity of the accident, so as to provide guidance for rescue operations.

Assume that there is a freeway accident involving *n* non-human factors and *m* human factors. The accidents are divided into three categories: death, injury, and property damage. Therefore, the evaluation set for this accident is V={death,injury,propertydamage}. The number of accidents in terms of death, injury, and property damage are respectively $h_{1},h_{2},h_{3}$. The final evaluation severity *β* can be selected using the maximum membership principle in each category. The true severity level is set as $\beta^{⁎}$. In the scenario where there are three accidents of varying severity, $g_{1},g_{2}$ and $g_{3}$ denote the damage of the death, injury, and property, respectively. We assume that the test data set contains an equal number of occurrences for both *β* and $\beta^{⁎}$. $\alpha_{i}$ represents the accuracy of predicting accidents with different severity levels, where $\alpha_{i}=g_{i}/h_{i}⁎100\text{\%},i=1,2,3$. The comprehensive accuracy is defined as $\alpha^{⁎}=\sum_{i=1}^{3}\alpha_{i}⁎\frac{h_{i}}{\sum_{i=1}^{3}h_{i}}$. Table 1 presents the mathematical symbols and their explanations to be used.

**Table 1:** Common symbols and definitions.

Notation	Definition
*P*	The data set of traffic accidents is available
*P*_*c*_	The data of the current accident is available
*U*	The set of factors derived from the data in *P*
*U*_*k*_	The *k*-th level factor
$u_{n_{i}}^{\left(i\right)}$	The *n*_*i*_-th second-level factor under the *i*-th first-level factor
*V*	The evaluation set
*v*_*m*_	The m-th evaluation in *V*
*R*	The overall fuzzy evaluation matrix
*R*_*i*_	The overall fuzzy evaluation matrix of *U*_*i*_
*W*	The weight set of *U*_1_ to *U*_*k*_
*W*_*i*_	The weight set of $u_{1_{i}}^{\left(i\right)}$ to $u_{n_{i}}^{\left(i\right)}$
*B*	The evaluation result set of *U*
*B*_*i*_	The evaluation result set of *U*_*i*_
*β*	The severity of the accident calculated through *B*
*β*^⁎^	The actual severity of the accident
*α*_*i*_	The accuracy of predicting accidents with different severity
*α*^⁎^	The comprehensive accuracy of *α*_*i*_

### 3.2 Two-level fuzzy comprehensive evaluation

The steps for the two-level fuzzy comprehensive evaluation method are provided as follows.

1. The set of factor set $U=\{u_{1},u_{2},\cdots ,u_{n}\}$ is divided into several groups, resulting in $U=\{U_{1},U_{2},\cdots ,U_{k}\}$.
  Where $U={⋃}_{i=1}^{k}U_{i},U_{i}\cap U_{j}=\Phi (i\neq j)$, $U=\{U_{1},U_{2},\cdots ,U_{k}\}$ is referred to as the first factor set.
2. Evaluation set $V=\{v_{1},v_{2},\cdots ,v_{m}\}$ is given. First, the $n_{i}$ factors in the factor set $U_{i}=\{u_{1}^{\left(i\right)},u_{2}^{\left(i\right)},\cdots ,u_{n_{i}}^{\left(i\right)}\}$ are evaluated individually based on their ratios in terms of three different accident severity: death, injury, and property damage. This evaluation process results in a single-factor evaluation matrix
  $R_{i}=\left(\begin{array}{cccc} r_{11}^{\left(i\right)} & r_{12}^{\left(i\right)} & \cdots & r_{1m}^{\left(i\right)}\\ r_{21}^{\left(i\right)} & r_{22}^{\left(i\right)} & \cdots & r_{2m}^{\left(i\right)}\\ \vdots & \vdots & \ddots & \vdots \\ r_{n_{i}1}^{\left(i\right)} & r_{n_{i}2}^{\left(i\right)} & \cdots & r_{n_{i}m}^{\left(i\right)} \end{array}\right)$
  The weight of the factor set $U=\{U_{1},U_{2},\cdots ,U_{k}\}$ is computed as $W=\{W_{1},W_{2},\cdots ,W_{k}\}$. The weight of the factor set $U_{i}=\{u_{i}^{\left(i\right)},u_{2}^{\left(i\right)},\cdots ,u_{n_{i}}^{\left(i\right)}\}$ is computed as $W_{i}=(w_{i}^{\left(i\right)},w_{2}^{\left(i\right)},\cdots ,w_{n_{i}}^{\left(i\right)})$.
3. The two-level factor evaluation matrix is computed as $B_{i}=W_{i}\circ R_{i},i=\{1,2,3,\cdots ,k\}$. The overall evaluation matrix for two-level factors is
  $R=\left(\begin{array}{c} B_{1}\\ B_{2}\\ \vdots \\ B_{k} \end{array}\right)$
  The final evaluation result is B=A∘R. The *β* is selected from *B* based on the principle of maximum membership.

### 3.3 Problem description

When a freeway accident occurs, it is crucial to accurately predict the severity of the accident based on accident information. There is an urgent need to study a method for predicting the severity of freeway accidents using two-level fuzzy comprehensive evaluation. This method aims to fully consider the influencing factors of accidents and the fuzzy relationships between these factors, in order to achieve quick, accurate, and reliable prediction of accident severity. The freeway accident prediction method designed in this paper, which is based on a two-level fuzzy comprehensive evaluation, satisfies the following requirements:

• **Accuracy**: The proposed freeway accident prediction method has higher accuracy than the existing methods.
• **Efficiency**: The proposed freeway accident prediction method has a smaller computational cost than the existing methods.

## 4 The proposed FASP method

### 4.1 Factor preparation

The factor sets are determined as follows. The accident factors are divided into two categories: human factors and non-human factors. The factor set $U=\{U_{1},U_{2}\}$, which denotes the sets of non-human and human factors, respectively. There are 36 human factors and 6 non-human factors. The non-human factors $U_{1}$ include accident time, driving experience, weather conditions, vehicle type, road conditions, and collision type. The human factor set $U_{2}$ includes four major categories: motor vehicle violations, motor vehicle non-violation faults, non-motor vehicle violations, and pedestrian violations.

In particular, motor vehicle violations include overspeeding, drunk driving, wrong-way driving, fatigued driving, illegal lane change, illegal overtaking, illegal reversing, illegal U-turn, illegal meeting, illegal towing, illegal jaywalking, illegal driving on the road, illegal parking, illegal lane usage, illegal loading, illegal loading exceeding limits and dangerous goods transportation, violation of traffic signals, failure to yield as required, driving without a license, improper use of lights, and other unsafe behaviors. Motor vehicle non-violation faults include improper braking, improper steering, improper throttle control, and other improper operations. Non-motor vehicle violations include wrong-way riding, illegal driving on the road, illegal lane usage, violation of traffic signals, failure to yield as required, and other unsafe behaviors. Pedestrian violations include illegal road crossing, illegal occupation of roadway, violation of traffic signals, and other unsafe behaviors.

In order to facilitate factor processing, it is necessary to encode the original dataset. Table 2 shows the coding for each of the identified factors. The study's data is publicly accessible on GitHub via the repository located at https://github.com/My-Belief/prediction-of-freeway-accident-severity. It can be seen that this study chooses to encode human factors as a whole. Non-human factors are encoded based on the specific number of categories in the two-level factors.

**Table 2:** Coding table of factors affecting accidents.

One-level	Two-level	Category	Code
Non-human Factors	Accident time	Daylight	1
		Night	2
	Driving experience	<3	1
		3 − 10	2
		>10	3
	Weather	Sunny	1
		Adverse weather	2
	Vehicle type	Passenger car	1
		Cars	2
		Cargo	3
	Road	Dry	1
		Wet	2
		Snow	3
		Water accumulation	4
	Collision type	Frontal collision	1
		Side collision	2
		Rear-end collision	3
Human factors	Motor vehicle violations	Overspeeding	1
		Drunk driving	2
		Wrong-way driving	3
		Fatigued driving	4
		Illegal lang change	5
	⋮	⋮	⋮
		Failure to yield as required	31
		Other unsafe behaviors	32
	Pedestrian Violations	Illegal road crossing	33
		Illegal occupation of the road	34
		Violation of traffic signal	35
		Other unsafe behaviors	36

### 4.2 Evaluation matrix calculation

The evaluation matrix is obtained by using membership degree formulas. The membership degree matrix is calculated based on the proportions of the factor at different levels in the *P* and $P_{c}$. Assume that the proportions of a certain factor in terms of death, injury, and property damage are $\alpha_{1}$, $\alpha_{2}$, and $\alpha_{3}$, respectively. The evaluation set of this factor is $r_{1}$, $r_{2}$, and $r_{3}$, which satisfy the following conditions:

(1) $$\frac{r_{i}}{\sum_{i=1}^{3}r_{i}}=\frac{\alpha_{i}}{\sum_{i=1}^{3}\alpha_{i}}$$

(2) $$\sum_{i=1}^{3}r_{i}=1$$

According to Equation (1) and Equation (2), the evaluation matrix $R_{1}$ is calculated for non-human factors in freeway accidents.

(3) $$R_{1}=\left(\begin{array}{ccc} r_{11}^{\left(1\right)} & r_{12}^{\left(1\right)} & r_{13}^{\left(1\right)}\\ r_{21}^{\left(1\right)} & r_{22}^{\left(1\right)} & r_{23}^{\left(1\right)}\\ \vdots & \vdots & \vdots \\ r_{n1}^{\left(1\right)} & r_{n2}^{\left(1\right)} & r_{n3}^{\left(1\right)} \end{array}\right)$$

Similarly, according to Equation (1) and Equation (2), the evaluation matrix $R_{2}$ for human factors is calculated.

(4) $$R_{2}=\left(\begin{array}{ccc} r_{11}^{\left(2\right)} & r_{12}^{\left(2\right)} & r_{13}^{\left(2\right)}\\ r_{21}^{\left(2\right)} & r_{22}^{\left(2\right)} & r_{23}^{\left(2\right)}\\ \vdots & \vdots & \vdots \\ r_{m1}^{\left(2\right)} & r_{m2}^{\left(2\right)} & r_{m3}^{\left(2\right)} \end{array}\right)$$

### 4.3 Weight set calculation

The weight set is divided into primary factor weights and two-level factor weights. The weights of the two-level factors are determined based on the *P* and $P_{c}$. The primary factors are divided into non-human factors and human factors. It is necessary to determine the weights of the primary factors and the weights of the two-level factors under the primary factors. The calculation process of the weight set using the entropy method is as follows. First, the data sets are normalized of the dataset to obtain the normalized dataset *P*. The normalization process is given as

(5) $$P_{ij}=\frac{C_{ij}}{\sum_{i=1}^{m}C_{ij}}$$

where $P_{ij}$ represents the element value in the normalized dataset *P* at the *i*-th row and *j*-th column. $C_{ij}$ refers to the corresponding element value in the dataset. The summation term in the denominator ensures that each column's elements sum up to 1. The normalized entropy $E_{j}$ is computed for each column of data. The entropy is calculated using the formula:

(6) $$E_{j}=-\sum_{i=1}^{m}P_{ij}\log(P_{ij})$$

where $E_{j}$ represents the normalized entropy for the *j*-th column. $P_{ij}$ indicates the element value in the normalized dataset *P* at the *i*-th row and *j*-th column. The logarithm is taken base 2 or natural logarithm depending on preference. The weight $w_{j}$ is determined for each column of data. The weight is calculated using the formula:

(7) $$w_{j}=\frac{1-E_{j}}{\sum_{j=1}^{n}(1-E_{j})}$$

where $w_{j}$ represents the weight value for the *j*-th column. $E_{j}$ denotes the normalized entropy for the *j*-th column. The summation in the denominator ensures that the weights sum up to 1. All the weights are normalized, so that their sum is equal to 1. The normalization formula for the weights is:

(8) $$w=\frac{w_{j}}{\sum_{j=1}^{n}w_{j}}$$

where the *w* represents the normalized value of each weight. $w_{j}$ signifies the weight value for the *j*-th column. The summation in the denominator ensures that the normalized weights sum up to 1. By inputting different data into equations (5)-(8), the corresponding weight sets can be calculated. When the input data consists only of the dataset for non-human factors, the weight values between each factor of non-human factors can be calculated as $W_{1}$.

(9) $$W_{1}=\left(w_{1}^{1},w_{2}^{1},\cdots ,w_{n}^{1}\right)$$

Similarly, using the dataset for human factors, the weight values for each factor of human factors can be obtained as $W_{2}$. By using the complete dataset, the weight values for both human factors and non-human factors can be obtained as *W*, which is given as follows.

(10) $$W_{2}=\left(w_{1}^{2},w_{2}^{2},\cdots ,w_{m}^{2}\right)$$

(11) $$W=\left(W_{U_{1}},W_{U_{2}}\right)$$

### 4.4 Evaluation result calculation

According to Equation (3) and Equation (9), the matrix evaluation matrix $B_{1}$ for non-human factor is

(12) $$B_{1}=W_{1}\circ R_{1}=\left(\begin{array}{ccc} b_{11}^{1} & b_{12}^{1} & b_{13}^{1} \end{array}\right)$$

According to Equation (4) and Equation (10), the matrix evaluation matrix $B_{2}$ for human factor is

(13) $$B_{2}=W_{2}\circ R_{2}=\left(\begin{array}{ccc} b_{11}^{2} & b_{12}^{2} & b_{13}^{2} \end{array}\right)$$

According to Equation (11)-(13), the final evaluation matrix *B* is

(14) $$B=W\circ \;R=W\circ \;\left(\begin{array}{c} B_{1}\\ B_{2} \end{array}\right)$$

According to the calculation of *B* in Equation (14), the severity of the freeway accident is determined based on the maximum membership principle. The algorithm for predicting the severity of freeway accidents based on two-level fuzzy comprehensive evaluation is shown in Algorithm 1.

**Algorithm 1:**
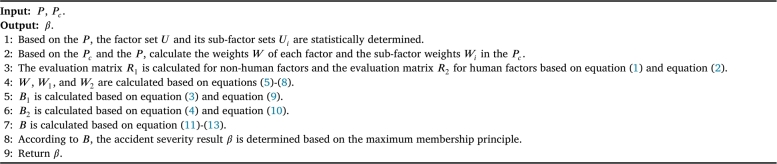
The proposed FASP method.

## 5 Experimental setup and results

### 5.1 Setup

The experiment was conducted on a computer with an AMD R7 5800H @ 3.20Hz processor and 16GB RAM. Python was used for extensive simulations. The datasets used in this experiment come from the transportation professional knowledge service system in 2016 [62] and US highway railroad crossing accident [63]. This system encompasses six major categories of resources, including scientific literature, research foundations, statistical data, engineering construction data, management decision data, and other resources, totaling 38 datasets with millions of records. It covers 175 professional fields, including freeway engineering, bridge engineering, tunnel engineering, transportation engineering, port and waterway engineering, road transportation, water transportation, comprehensive transportation, urban public transportation, automotive engineering, shipbuilding engineering, transportation planning and management, transportation economics, transportation safety, green transportation, and intelligent transportation. Among them, a total of 180 freeway accident records from 2005 to 2016 were included. The US dataset is taken from the US Department of Transportation. The data is provided by the FRA Office of Railroad Safety and the dataset owner is Jared McCulloch. The dataset covers accidents from 1st January 1975 to 28 February 2021. It has 239487 rows and 141 columns. This data is extracted from the accident report form. The main features consist of Geography, time frame, type of crossing, type of accident, type of vehicle, type of highway user, type of equipment, and highway User action.

FASP will compare the performance of various algorithms such as DNN [30], Logistic Regression (LR) [20], [23], [41], Adaboost (ADA) [64], Random Forest (RF) [20], [23], [21], Fuzzy C-means (FCM) [20], [65], K-Nearest Neighbors (KNN) [66], Neural Network (NN) [67], Support Vector Machine (SVM) [28] and BAYESIAN (BAYES) [23] in terms of prediction accuracy ($\alpha_{i}$), comprehensive accuracy ($\alpha^{⁎}$), and computational cost. Simultaneously, the study will investigate the influence of the number of factors considered in the two-level fuzzy comprehensive evaluation. Through experiments, it aims to demonstrate the impact of the number of considered factors on prediction accuracy.

In order to study the performance of the proposed method under different types of freeways and different traffic flow conditions, 15 different freeway accident severity distributions were set up. As different freeways have different distributions of accident data, Table 3 lists the proportional distribution of death, injury, and property damage for 15 data sets. For example, S1 denotes the data distribution in an accident-prone area with a large traffic flow. The proportion of fatal accidents is 0.6, the proportion of injuries is 0.2, and property damage accidents accounted for 0.2. S15 denotes the proportion of accidents of different severity on roads with small traffic volumes and good road conditions. S7 denotes the proportion of accidents of different severity in rainy and snowy weather. In this experiment, a total of 1800 data samples were synthesized for each training set S1-S15 based on the aforementioned proportional distribution. This experiment also considered different data quantities and synthesized training set datasets with quantities of 3000, 5000, 7000, and 9000, respectively. Among them, the ratio of accidents involving death, injury, and property damage was 1:1:1. For the aforementioned training sets, the number of testing sets is set to be 1/9 of the corresponding training set size. Additionally, the impact of the number of factors on prediction accuracy was considered and corresponding experiments were conducted.

**Table 3:** Accident data distribution ratio setting.

Severity	S1	S2	S3	S4	S5	S6	S7	S8	S9	S10	S11	S12	S13	S14	S15
death	0.6	0.5	0.4	0.3	0.2	0.5	0.4	0.3	0.2	0.4	0.3	0.2	0.3	0.2	0.2
injury	0.2	0.3	0.4	0.5	0.6	0.2	0.3	0.4	0.5	0.2	0.3	0.4	0.2	0.3	0.2
property	0.2	0.2	0.2	0.2	0.2	0.3	0.3	0.3	0.3	0.4	0.4	0.4	0.5	0.5	0.6

### 5.2 Results

#### 5.2.1 Prediction accuracy with different training sets

The method proposed has been experimentally demonstrated to have high accuracy in predicting the severity of freeway accidents. Table 4, Table 5, Table 6, Table 7, Table 8, Table 9, Table 10, Table 11, Table 12, Table 13 present the accuracy of the proposed FASP method, as well as the DNN, LR, ADA, RF, ADA, FCM, KNN, NN, SVM, and BAYES methods, in predicting death, injury and property damage, along with the comprehensive accuracy. These tables correspond to the severe accident prediction results under Chinese traffic accident datasets and US traffic accident datasets when the number of training sets is 1800, 3000, 5000, 7000, and 9000, respectively.

**Table 4:** Accuracy of prediction on a training set of 1800 in China.

Methods	Death	Injury	Property	Comprehensive Accuracy
FASP	68.00	77.50	69.00	71.50
DNN	58.40	77.75	58.80	64.98
LR	60.00	37.00	60.00	52.33
RF	59.30	82.45	63.55	68.43
ADA	45.50	100.00	50.00	65.17
FCM	45.50	75.00	66.50	62.33
KNN	60.00	82.50	61.00	67.83
NN	61.05	83.00	61.60	68.55
SVM	45.50	74.00	68.50	62.67
BAYES	47.00	95.76	40.00	60.92

**Table 5:** Accuracy of prediction on a training set of 1800 in the US.

Methods	Death	Injury	Property	Comprehensive Accuracy
FASP	87.00	81.00	85.50	84.50
DNN	48.96	51.28	50.33	50.19
LR	75.50	72.00	67.50	71.67
RF	87.50	84.60	80.50	84.20
ADA	84.00	83.50	82.50	83.33
FCM	61.00	66.00	87.00	71.33
KNN	54.50	34.50	37.00	42.00
NN	57.11	84.00	69.00	70.04
SVM	22.00	15.50	93.00	43.50
BAYES	87.00	84.50	79.50	83.67

**Table 6:** Accuracy of prediction on a training set of 3000 in China.

Methods	Death	Injury	Property	Comprehensive Accuracy
FASP	78.68	74.77	66.37	73.27
DNN	63.39	80.78	61.02	68.40
LR	62.76	37.84	61.56	54.05
RF	62.25	83.27	64.08	69.87
ADA	47.15	100.00	51.05	66.07
FCM	47.15	74.77	64.86	62.26
KNN	57.67	84.67	60.33	67.56
NN	63.20	82.00	67.86	71.02
SVM	45.00	74.33	67.33	62.22
BAYES	46.33	97.13	42.33	61.93

**Table 7:** Accuracy of prediction on a training set of 3000 in the US.

Methods	Death	Injury	Property	Comprehensive Accuracy
FASP	88.33	87.00	90.00	88.44
DNN	88.00	84.33	51.73	74.69
LR	69.67	89.00	67.33	75.33
RF	80.67	88.67	86.00	85.11
ADA	87.30	93.30	84.40	88.33
FCM	59.67	60.33	86.67	68.89
KNN	53.33	41.33	38.33	44.33
NN	65.00	60.23	59.19	61.47
SVM	24.67	15.00	91.33	43.67
BAYES	85.67	87.33	82.33	85.11

**Table 8:** Accuracy of prediction on a training set of 5000 in China.

Methods	Death	Injury	Property	Comprehensive Accuracy
FASP	79.46	76.40	67.93	74.60
DNN	64.13	89.19	61.01	71.44
Logistic	63.96	39.64	60.18	54.59
RF	65.30	84.25	67.24	72.26
AdaBoost	47.03	99.64	54.05	66.91
FCM	47.03	75.14	65.05	62.41
KNN	64.80	85.80	64.00	71.53
NN	67.00	86.60	61.20	71.60
SVM	45.80	74.60	69.20	63.20
BAYES	46.60	97.32	44.00	62.64

**Table 9:** Accuracy of prediction on a training set of 5000 in the US.

Methods	Death	Injury	Property	Comprehensive Accuracy
FASP	89.20	76.00	92.20	85.80
DNN	78.20	62.40	67.40	69.33
LR	68.80	74.00	72.20	71.67
RF	78.60	81.60	86.60	82.27
ADA	41.20	95.20	86.00	74.13
FCM	57.80	60.20	87.80	68.60
KNN	59.40	39.80	41.00	46.73
NN	67.52	73.74	67.14	69.47
SVM	17.00	23.80	82.00	40.93
BAYES	87.60	72.80	81.20	80.53

**Table 10:** Accuracy of prediction on a training set of 7000 in China.

Methods	Death	Injury	Property	Comprehensive Accuracy
FASP	81.21	76.96	67.57	75.25
DNN	67.59	89.64	58.35	71.86
LR	65.64	41.06	62.29	56.33
RF	68.23	86.54	67.36	74.04
ADA	48.39	99.36	54.17	67.31
FCM	48.39	76.58	66.28	63.75
KNN	66.14	84.14	66.43	72.24
NN	67.67	84.43	60.28	70.79
SVM	47.86	75.57	68.00	63.81
BAYES	46.00	98.17	43.57	62.58

**Table 11:** Accuracy of prediction on a training set of 7000 in the US.

Methods	Death	Injury	Property	Comprehensive Accuracy
FASP	88.57	83.14	94.00	88.57
DNN	80.57	95.14	87.00	87.57
LR	84.57	75.29	70.71	76.86
RF	79.29	88.14	86.57	84.67
ADA	88.43	78.57	87.86	84.95
FCM	55.71	61.71	88.43	68.62
KNN	60.00	39.00	44.00	47.67
NN	76.05	74.14	76.06	75.42
SVM	19.43	31.00	61.86	37.43
BAYES	88.29	76.57	83.29	82.72

**Table 12:** Accuracy of prediction on a training set of 9000 in China.

Methods	Death	Injury	Property	Comprehensive Accuracy
FASP	80.30	77.80	67.10	75.07
DNN	67.28	92.70	60.11	73.36
LR	65.20	40.10	63.40	56.23
RF	67.98	87.59	66.50	74.02
ADA	49.39	99.40	54.30	67.70
FCM	49.40	77.30	66.00	64.23
KNN	64.71	85.14	69.14	73.00
NN	67.15	89.56	62.69	73.13
SVM	48.33	76.33	69.56	64.74
BAYES	45.89	98.23	43.67	62.59

**Table 13:** Accuracy of prediction on a training set of 9000 in the US.

Methods	Death	Injury	Property	Comprehensive Accuracy
FSAP	92.33	89.77	92.11	91.40
DNN	88.89	78.11	92.44	86.48
LR	75.56	91.78	74.78	80.71
RF	85.56	88.22	85.22	86.33
ADA	91.22	94.33	81.56	89.04
FCM	58.89	63.11	88.44	70.15
KNN	59.56	40.11	46.44	48.70
NN	74.54	79.25	82.28	78.69
SVM	19.00	30.89	54.11	34.67
BAYES	87.11	79.33	82.0	82.81

As shown in Table 4, the proposed method achieves an accuracy of 68.00% in predicting deadly accidents, which is significantly higher than DNN, LR, RF, ADA, FCM, KNN, NN, SVM, and BAYES. In terms of predicting injury accidents, the accuracy of the proposed method reaches 77.50%, lower than DNN, ADA, KNN, NN, RF, and BAYES, but higher than LR FCM and SVM. However, the accuracy of the proposed method is higher than DNN, ADA, KNN, NN, RF, and BAYES in predicting deadly accidents and property damage accidents. Regarding the prediction accuracy of property damage accidents, the proposed method achieves an accuracy of 69.00%. The accuracy rate of predicting property damage accidents is 71.50%, which is higher than the prediction accuracy of DNN, LR, ADA, RF, ADA, FCM, KNN, NN, SVM, and BAYES for property damage accidents.

As shown in Table 5, the proposed method achieves an accuracy of 87.00% in predicting deadly accidents, which is significantly higher than DNN, Logistic, ADA, FCM, KNN, NN, and SVM. In terms of predicting injury accidents, the accuracy of the proposed method reaches 81.00%, lower than RF, ADA, NN, and BAYES, but higher than DNN, LR, FCM, KNN, and SVM. However, the accuracy of the proposed method is higher than RF, ADA, NN, and BAYES in predicting deadly accidents and property damage accidents. Regarding the prediction accuracy of property damage accidents, the proposed method achieves an accuracy of 85.50%. The accuracy rate of predicting property damage accidents is 84.50%, which is higher than the prediction accuracy of DNN, LR, ADA, RF, ADA, FCM, KNN, NN, SVM, and BAYES for property damage accidents.

As shown in Table 6 on a training set of 3000 accidents in Chinese traffic accident datasets, the proposed FASP method achieves an accuracy of 78.68% and 66.37% in predicting deadly accidents and property damage accidents, respectively. The accuracy of the proposed method for both severity types is higher than DNN, LR, ADA, RF, ADA, FCM, KNN, NN, SVM, and BAYES. In predicting injury accidents, the accuracy reaches 74.77%, which is lower than DNN, RF, ADA, KNN, and BAYES, but higher than LR and SVM. However, the accuracy of the proposed method is higher than DNN, RF, ADA, KNN, and BAYES in predicting deadly accidents and property damage accidents. Furthermore, the comprehensive accuracy of the proposed method is 73.27%, which is higher than DNN, LR, ADA, RF, ADA, FCM, KNN, NN, SVM, and BAYES.

As shown in Table 7 on a training set of 3000 accidents in US traffic accident datasets, the proposed FASP method achieves an accuracy of 88.33% and 90.00% in predicting deadly accidents and property damage accidents, respectively. The accuracy of the proposed method for both severity types is higher than DNN, LR, ADA, RF, ADA, FCM, KNN, NN, SVM, and BAYES. In predicting injury accidents, the accuracy reaches 74.77%, which is lower than LR, RF, ADA, and BAYES, but higher than DNN, FCM, KNN, NN, and SVM. Furthermore, the comprehensive accuracy of the proposed method is 88.244%, which is higher than DNN, LR, ADA, RF, ADA, FCM, KNN, NN, SVM, and BAYES.

As shown in Table 8 on a training set of 5000 accidents in Chinese traffic accident datasets, the proposed method achieves an accuracy of 79.46% and 67.93% in predicting deadly accidents and property damage accidents, respectively. The accuracy of the proposed method for both severity types is higher than DNN, LR, ADA, RF, ADA, FCM, KNN, NN, and BAYES. In predicting injury accidents, the accuracy reaches 76.40%, which is lower than the accuracy of DNN, RF, ADA, KNN, NN, and BAYES methods in predicting deadly accidents, but higher than the accuracy of LR, FCM, and SVM methods in predicting injury accidents. However, the accuracy of DNN, RF, ADA, KNN, NN, and BAYES methods in predicting deadly accidents and property damage accidents is lower than that of the proposed method. Additionally, the comprehensive accuracy of the proposed method is 74.60%, which is higher than DNN, LR, ADA, RF, ADA, FCM, KNN, NN, SVM, and BAYES.

As shown in Table 9 on a training set of 5000 accidents in US traffic accident datasets, the proposed method achieves an accuracy of 89.20% and 92.20% in predicting deadly accidents and property damage accidents, respectively. The accuracy of the proposed method for both severity types is higher than DNN, LR, ADA, RF, ADA, FCM, KNN, NN, SVM, and BAYES. In predicting injury accidents, the accuracy reaches 76.00%, which is lower than the accuracy of RF and ADA methods in predicting deadly accidents, but higher than the accuracy of DNN, LR, ADA, FCM, KNN, NN, SVM, and BAYES methods in predicting injury accidents. However, the accuracy of RF and ADA methods in predicting deadly accidents and property damage accidents is lower than that of the proposed method. Additionally, the comprehensive accuracy of the proposed method is 85.80%, which is higher than DNN, LR, ADA, RF, ADA, FCM, KNN, NN, SVM, and BAYES.

As shown in Table 10 on a training set of 7000 accidents in Chinese traffic accident datasets, the proposed method achieves an accuracy of 81.21% and 67.57% in predicting deadly accidents and property damage accidents, respectively. The accuracy of the proposed method for both severity types is higher than DNN, LR, ADA, RF, ADA, FCM, KNN, NN, SVM, and BAYES. In predicting injury accidents, the accuracy reaches 76.96%, which is lower than the accuracy of DNN, RF, ADA, KNN, NN, and BAYES methods in predicting injury accidents, but higher than the accuracy of LR, SVM, and FCM methods in predicting injury accidents. However, the accuracy of DNN, RF, ADA, KNN, NN, and BAYES methods in predicting deadly accidents and property damage accidents is lower than that of the proposed method. Additionally, the comprehensive accuracy of the proposed method is 75.25%, which is higher than DNN, LR, ADA, RF, ADA, FCM, KNN, NN, SVM, and BAYES.

As shown in Table 11 on a training set of 7000 accidents in US traffic accident datasets, the proposed method achieves an accuracy of 88.57% and 94.00% in predicting deadly accidents and property damage accidents, respectively. The accuracy of the proposed method for both severity types is higher than DNN, LR, ADA, RF, ADA, FCM, KNN, NN, SVM, and BAYES. In predicting injury accidents, the accuracy reaches 83.14%, which is lower than the accuracy of DNN and RF methods in predicting injury accidents, but higher than the accuracy of LR, ADA, ADA, FCM, KNN, NN, SVM, and BAYES methods in predicting injury accidents. However, the accuracy of DNN and RF methods in predicting deadly accidents and property damage accidents is lower than that of the proposed method. Additionally, the comprehensive accuracy of the proposed method is 88.57%, which is higher than DNN, LR, ADA, RF, ADA, FCM, KNN, NN, SVM, and BAYES.

As shown in Table 12 on a training set of 9000 accidents in Chinese traffic accident datasets, the proposed method achieves an accuracy of 80.30% and 67.10% in predicting deadly accidents and property damage accidents, respectively. The accuracy of the proposed method for both severity types is higher than DNN, LR, ADA, RF, ADA, FCM, KNN, NN, and BAYES. In predicting injury accidents, the accuracy reaches 77.80%, which is lower than the accuracy of DNN, RF, ADA, KNN, NN, and BAYES methods in predicting injury accidents, but higher than the accuracy of LR, FCM, and SVM methods in predicting injury accidents. However, the accuracy of DNN, RF, ADA, KNN, NN, and BAYES methods in predicting deadly accidents and property damage accidents is lower than that of the proposed method. Additionally, the comprehensive accuracy of the proposed method is 75.07%, which is DNN, LR, ADA, RF, ADA, FCM, KNN, NN, SVM, and BAYES.

As shown in Table 13 on a training set of 9000 accidents in US traffic accident datasets, the proposed method achieves an accuracy of 92.33% and 92.11% in predicting deadly accidents and property damage accidents, respectively. The accuracy of the proposed method for both severity types is higher than LR, ADA, RF, ADA, FCM, KNN, NN, SVM, and BAYES. In predicting injury accidents, the accuracy reaches 89.77%, which is lower than the accuracy of LR and ADA methods in predicting injury accidents, but higher than the accuracy of DNN, RF, ADA, FCM, KNN, NN, SVM, and BAYES methods in predicting injury accidents. However, the accuracy of LR and ADA methods in predicting deadly accidents and property damage accidents is lower than that of the proposed method. Additionally, the comprehensive accuracy of the proposed method is 91.40%, which is higher than DNN, LR, ADA, RF, ADA, FCM, KNN, NN, SVM, and BAYES. In summary, the proposed FASP method has a superior comprehensive accuracy under various data conditions, indicating that the proposed method has a higher overall predictive ability compared to other methods.

#### 5.2.2 Prediction accuracy with different data distribution

Different data distributions correspond to different road types and traffic volumes. The comprehensive accuracy rates are shown using experiments based on the different accident data distribution ratios presented in Table 3. Table 14 present the comprehensive accuracy rates of the proposed method, DNN, LR, ADA, RF, ADA, FCM, KNN, NN, SVM, and BAYES for data distributions S1-S15. Taking S1 as an example, through experiments and calculations, it was found that the proposed method achieved a comprehensive accuracy rate of 69.66%, which is higher than DNN, LR, ADA, RF, ADA, FCM, KNN, NN, SVM, and BAYES.

**Table 14:** The comprehensive accuracy for S1-S15 on the traffic accident datasets in China.

	S1	S2	S3	S4	S5	S6	S7	S8	S9	S10	S11	S12	S13	S14	S15
FASP	69.66	72.13	74.60	77.07	79.54	69.62	72.09	74.56	77.03	69.58	72.05	74.52	69.54	72.01	69.50
DNN	30.34	28.83	27.32	25.81	24.30	33.86	32.35	30.84	29.33	37.38	35.89	34.36	40.90	39.39	33.41
LR	54.22	58.24	62.26	66.28	70.30	54.76	58.78	62.80	66.82	55.30	59.32	63.34	55.84	59.86	56.38
RF	68.86	70.93	73.01	75.09	77.17	68.58	70.66	72.74	74.82	68.30	70.38	72.46	68.03	70.11	67.76
ADA	63.20	66.68	70.16	73.64	77.11	62.08	65.56	69.03	72.51	60.95	64.43	67.91	59.83	63.30	58.70
FCM	65.31	65.47	65.63	65.79	65.94	63.06	63.22	63.38	63.54	60.81	60.97	61.13	58.56	58.72	56.31
KNN	69.30	68.65	70.40	73.10	76.60	66.25	66.85	68.80	73.25	66.40	67.65	72.10	67.95	71.09	68.20
NN	68.23	70.35	71.82	74.74	77.77	66.34	67.96	70.16	74.41	65.85	68.14	71.03	67.18	68.73	69.39
SVM	68.60	66.35	63.00	63.20	69.00	63.95	63.25	63.65	66.55	64.00	63.25	66.00	59.20	65.45	63.90
BAYES	58.10	62.55	68.00	72.30	77.60	55.55	61.15	66.10	71.65	55.20	59.90	66.50	54.90	61.55	56.80

Table 15 present the comprehensive accuracy rates of the proposed method, DNN, LR, ADA, RF, ADA, FCM, KN, NN, SVM, and BAYES for data distributions S1-S15. Taking S15 as an example, through experiments and calculations, it was found that the proposed method achieved a comprehensive accuracy rate of 87.50%, which is higher than DNN, LR, ADA, RF, ADA, FCM, KNN, NN, SVM, and BAYES. The FASP method considers the fuzzy relationship between factors by utilizing membership degrees, which leads to a more comprehensive consideration of factors when predicting the severity of freeway accidents. In contrast, methods such as DNN, LR, ADA, RF, ADA, FCM, KNN, NN, SVM, and BAYES have less sufficient consideration of the relationships between factors. This may result in higher prediction accuracy for categories with larger data amounts, but lower prediction accuracy for categories with smaller data amounts, especially in situations where data is insufficient or imbalanced. Although the FASP method is also influenced by the dataset, the impact is smaller compared to the aforementioned methods. As a result, the comprehensive prediction accuracy of the FASP method is higher than that of the mentioned methods.

**Table 15:** The comprehensive accuracy for S1-S15 on the traffic accident datasets in the US.

	S1	S2	S3	S4	S5	S6	S7	S8	S9	S10	S11	S12	S13	S14	S15
FASP	75.30	87.80	86.50	84.36	85.60	84.20	86.70	81.85	83.85	87.70	84.70	84.50	88.20	82.10	87.50
DNN	28.70	59.73	42.46	50.95	60.49	56.02	46.87	43.45	56.98	52.22	48.98	60.73	53.09	52.01	64.17
LR	53.50	72.50	72.00	69.60	69.40	71.25	73.55	72.90	76.70	73.10	74.00	69.10	65.85	71.15	65.80
RF	55.40	87.79	83.88	81.34	82.01	83.15	83.90	81.62	76.33	85.42	82.73	81.13	85.83	75.84	85.98
ADA	41.50	85.75	30.20	75.70	84.30	80.55	75.65	81.70	73.10	72.00	68.40	80.80	84.75	68.75	83.40
FCM	69.50	67.85	69.70	71.55	73.40	66.90	68.75	70.60	72.45	67.80	69.65	47.50	68.70	70.40	68.60
KNN	30.50	50.85	45.80	46.30	53.40	54.85	46.40	44.40	46.35	50.70	44.15	44.60	51.85	48.75	55.60
NN	46.55	67.41	66.76	54.06	60.58	54.70	55.89	48.26	64.80	59.20	59.36	52.80	54.61	52.98	60.23
SVM	45.40	49.40	46.40	50.00	60.00	49.75	39.25	43.05	50.00	43.40	39.55	37.60	48.25	47.05	58.30
BAYES	73.50	80.60	81.20	83.25	83.60	79.10	78.90	80.85	82.85	78.90	80.80	81.60	80.80	80.50	73.10

#### 5.2.3 Prediction accuracy with different numbers of factors

**Figure 2:**
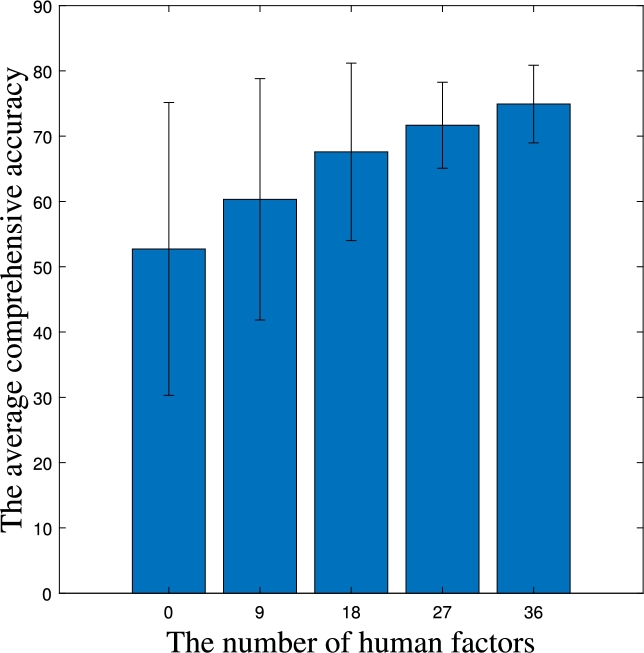
Average and variance of comprehensive accuracy.

To investigate the impact of the number of factors on prediction accuracy, the prediction accuracy is calculated for three different severity levels of accidents: death, injury, and property damage, with a data volume of 3000 and data distribution ranging from S1 to S15. The experiments focus on considering different numbers of human-related factors as an example. As shown in Fig. 2, the x-axis represents the number of considered factors, and the bar graph represents the average values ($\overset{¯}{\alpha^{⁎}}$) of the comprehensive accuracy and variances for S1 to S15 when considering different numbers of human-related factors. As the number of factors increases, the average value of comprehensive accuracy gradually increases. This means that the more factors are considered, the higher the prediction accuracy. It can also be observed that as the number of factors increases, the variance decreases. This implies that along with the improvement in accuracy, the prediction accuracy of accident severity also becomes more stable across different data distributions.

#### 5.2.4 Computational cost

**Figure 3:**
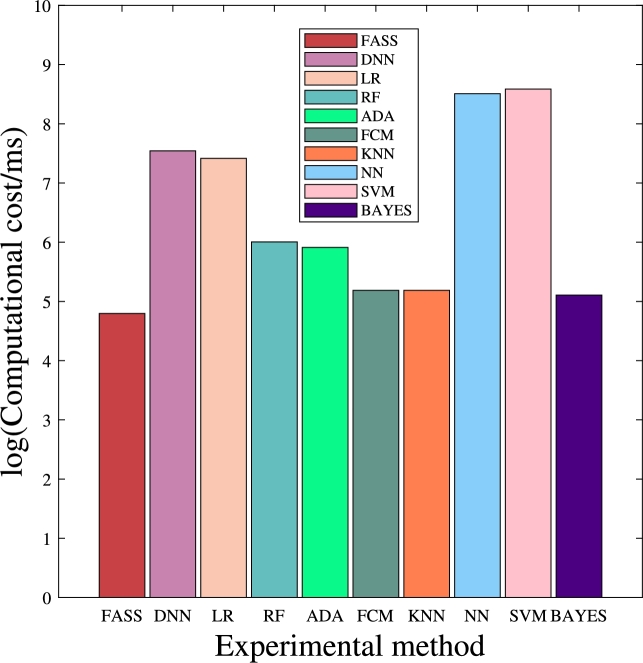
Computational cost.

The computational cost is used to evaluate the efficiency of the proposed FASP method. The computational costs of the DNN, LR, ADA, RF, ADA, FCM, KNN, NN, SVM, and BAYES algorithms were collected on training sets of 9000 and testing sets of 1000, respectively. The computational cost of predicting freeway accident severity is extremely important, as the rapid and accurate prediction of accident severity plays a vital role in safeguarding the lives and properties of accident victims. In this experiment, the computational cost includes data retrieval, training, and prediction processes. The FASP method, as depicted in Fig. 3, incurs a computational cost of 121 milliseconds, which is significantly smaller than that required by DNN, LR, ADA, RF, ADA, FCM, KNN, NN, SVM, and BAYES. This is attributed to the simplicity of matrix operations in FASP, eliminating the need for intricate training processes.

The proposed FASP method can be used for real-time accident severity prediction. Efficient and accurate severity prediction of a freeway accident can be achieved by the FASP method, which provides accident rescue guidance to reduce personnel and property losses. Existing work studied the real-time crash severity prediction of accidents [68], [69], [70]. The authors in [70] utilized spatial ensemble learning to predict the severity of an accident. The implementation of parallel computing utilizing a suite of 100 CPU cores resulted in a significant reduction of the total training and distillation time to approximately 3 minutes. The FASP method is calculated based on a two-level fuzzy comprehensive evaluation, which makes its calculation consumption extremely low. According to our experiments, the computational cost of the FASP method is 121 milliseconds, which is much smaller than that of the existing work. Therefore, the FASP method can provide real-time accident severity information and early warning of accident information, thus reducing the possibility of accidents.

## 6 Conclusion and future work

Freeway accident severity prediction is of great significance for accident prevention, road safety, and emergency rescue services in intelligent freeway systems. This paper investigates a novel problem of efficiently and accurately predicting the severity of freeway accidents to extend existing studies. Specifically, we collect the factors affecting freeway accidents to divide them into two levels. In the first level, the factors are divided into human factors and non-human factors. In the second level, 6 and 36 factors are further divided into human factors and non-human factors, respectively. Besides, we develop an efficient and accurate Freeway Accident Severity Prediction (FASP) method by utilizing a two-level fuzzy comprehensive evaluation. Based on the two-level factors, we determine the factor and evaluation sets to calculate the fuzzy evaluation matrix of a single factor. The entropy method is used to determine the weight matrix and the final evaluation matrix. We obtain the prediction of the severity of freeway accidents with the maximum membership principle. Additionally, the traffic accident datasets in China and the US are used to conduct experiments. The results show that the proposed FASP method has superior prediction accuracy and efficiency performance. In summary, this paper uses two-level fuzzy evaluation to assess the severity of the accident by comprehensively considering multiple influencing factors, thereby improving the accuracy of prediction. This multi-factor comprehensive evaluation method helps reduce the bias that may be caused by a single-factor evaluation. Accurate predictive models can help traffic authorities and emergency response teams better understand the severity of accidents, allowing them to make more effective resource allocation and emergency response decisions.

This paper investigates the problem of efficiently and accurately predicting the severity of freeway accidents using the two-level fuzzy comprehensive evaluation method. The evaluation and weight sets are calculated by using the data set. The key to improving the prediction accuracy lies in optimizing the evaluation and weight sets. In future research, we aim to improve the accuracy by optimizing the evaluation and weight sets. Specifically, we plan to combine the particle swarm optimization algorithm with the two-level fuzzy comprehensive evaluation method. The particle swarm optimization algorithm is utilized to determine the weight set in the two-level fuzzy comprehensive evaluation. The result of the two-level fuzzy comprehensive evaluation is taken as the objective function of the particle swarm algorithm. Furthermore, we will use techniques such as gradient boosting decision trees and support vector machines to obtain an initial set of weights, which helps the particle swarm algorithm conduct a more effective local optimization search based on multiple initial solutions.

## Declaration of generative AI and AI-assisted technologies in the writing process

During the preparation of this work, the authors used ChatGPT in order to improve readability. After using this service, the authors reviewed and edited the content as needed and took full responsibility for the content of the publication.

## CRediT authorship contribution statement

**Guanghui Wang:** Supervision, Project administration, Methodology, Funding acquisition. **Jinbo Li:** Writing – review & editing, Visualization, Validation, Software, Data curation. **Lingfeng Shen:** Visualization, Data curation. **Shuang Ding:** Writing – review & editing, Formal analysis. **Zongqi Shi:** Supervision, Resources, Investigation. **Fang Zuo:** Writing – review & editing, Methodology, Formal analysis, Conceptualization.

## Declaration of Competing Interest

The authors declare that they have no known competing financial interests or personal relationships that could have appeared to influence the work reported in this paper.

## Data Availability

The data used to support the findings of this study are accessible via the GitHub repository at https://github.com/My-Belief/prediction-of-freeway-accident-severity.
